# mTORC1 stimulates nucleotide synthesis through both transcriptional and post-translational mechanisms

**DOI:** 10.1186/2049-3002-2-S1-P6

**Published:** 2014-05-28

**Authors:** Issam Ben-Sahra, Stephane Ricoult, Jessica Howell, John Asara, Brendan Manning

**Affiliations:** 1 Harvard School of Public Health, Department of Genetics and Complex Diseases, Boston, MA, USA; 2 Division of Signal Transduction, Beth Israel Deaconess Medical Center and Department of Medicine, Harvard Medical School, Boston, MA, USA

## Background

Cellular growth signals stimulate anabolic processes. The mechanistic target of rapamycin (mTOR), as part of mTORC1, is a protein kinase that senses growth signals to regulate anabolic growth and proliferation. mTORC1 stimulates protein synthesis through effects on mRNA translation and ribosome biogenesis [1]. mTORC1 signaling also promotes *de novo* lipid and sterol synthesis through the activation of the sterol-response element-binding protein (SREBP) transcription factors, which stimulate the expression of the enzymes driving this biosynthetic process [2].

## Material and Methods

*TSC2 ^+/+^* MEFs, *TSC2 ^-/-^* MEFs, MCF10A expressing pBabe-empty vector or PI3KCA^H1047R^, HeLa cells, U87MG cell line were used in this study. To determine the relative levels of intracellular metabolites, extracts were prepared and analyzed by LC/MS/MS [3]. Regarding the U-^14^C-aspartate, U-14C-glycine incorporation into RNA and DNA, cells were serum starved for 15 hours and treated as indicated. Cells were harvested and RNA or DNA was isolated using Allprep DNA/RNA kits according to the manufacturer’s instructions and quantified using a spectrophotometer. For statistical analysis a two-tailed Student’s t-test was performed for all pairwise comparisons (*n*=3).

## Results

We find that activation of mTORC1 leads to the acute stimulation of metabolic flux through the *de novo* pyrimidine synthesis pathway [4]. We recently found that mTORC1 stimulates the *de novo* purine synthesis pathway. In contrast with pyrimidine synthesis, the regulation of the purine synthesis by mTORC1 signaling occurs through long-term mechanism. Indeed, we found that mTORC1 regulates the *de novo* purine synthesis pathway through the transcription factor SREBP.

## Conclusion

These findings demonstrate that growth signaling through mTORC1 promotes the production of new nucleotides to facilitate an increased demand for RNA and DNA. mTOR appears to be a central regulator of *de novo* nucleotide synthesis. Therefore, nucleotide synthesis joins protein and lipid synthesis as major anabolic processes stimulated by mTORC1 signaling.
